# A Hypothesis: The Interplay of Exercise and Physiological Heterogeneity as Drivers of Human Ageing

**DOI:** 10.3389/fphys.2021.695392

**Published:** 2021-09-09

**Authors:** Norman R. Lazarus, Stephen D. R. Harridge

**Affiliations:** Centre for Human & Applied Physiological Sciences, Faculty of Life Sciences & Medicine, King’s College London, London, United Kingdom

**Keywords:** ageing, exercise, health, physiology, lifestyle

## Abstract

As the inherent ageing process affects every facet of biology, physiology could be considered as the study of the healthy human ageing process. Where biological health is affected by lifestyle, the continual and continuing interaction of this process with physical activity and other lifestyle choices determine whether the ageing trajectory is toward health or disease. The presentation of both these states is further modified in individuals by the interaction of inherent physiological heterogeneity and the heterogeneity associated with responses and adaptions to exercise. The range of heterogeneity in healthy physiology is circumscribed by the necessity to conform to that of the human species. Our hypothesis is that, when sufficient exercise is present, these multiple interactions appear to produce an ageing profile that, while functional ability is in decline, remains synchronous, coherent, and integrated throughout most of life. In the absence of sufficient physical activity, physiology over time is gradually deteriorating toward the production of a lifestyle disease. Here, the ageing process, interacting with individual physiological heterogeneity, probably determines the age of presentation of a disease as well as the order of presentation of subsequent diseases. In this article, we discuss this hypothesis and related concepts in the context of the trajectory of healthy and non-healthy human ageing.

## Introduction

A common belief has been that “to grow old is to be sick” (Rowe and Kahn, 1999), but for a large segment of the older population, when the link between age and health decline is evaluated, chronological age is not a relevant marker for understanding, measuring, or experiencing healthy ageing (Yang and Lee, 2010; Lowsky et al., 2014).

The WHO defines healthy ageing as “the process of developing and maintaining the functional ability that enables wellbeing in older age” (WHO, 2015). While there are multiple definitions of healthy ageing, for the purposes of this article, we define it as being free of the diseases that are largely due to lifestyle influences. These lifestyle factors include physical inactivity/lack of exercise/sedentary behavior, poor nutrition, smoking and alcohol consumption, and which can result in cardiovascular disease, type II diabetes, and some cancers. However, healthy ageing can also be influenced by multiple factors including gender, race, income, educational level, and socioeconomic environment (Braveman, et al., 2011). In the arena circumscribed by diseases affiliated to lifestyle, this definition of healthy ageing encompasses the parameters as defined by the WHO definition of health (WHO, 2015). Healthy ageing is thus par excellence, reflected in the maintenance of whole-body integrated function (Lazarus and Harridge, 2017) despite an overall decline in physical capability and intrinsic capacity. In addition, while acknowledging that differences between definitions for “physical activity”/“inactivity”/“sedentary behavior” and “exercise” exist, for the purposes of this article, we have simply defined those that expend sufficient energy to be healthy as exercisers and those that do not as non-exercisers.

Many authors have drawn attention to the influence of lifestyle, particularly sufficient physical activity or exercise on the diseases that occur as people age (Fiaterone et al., 1990; Pedersen and Saltin, 2006, 2015; Booth et al., 2012; Lazarus and Harridge, 2017). The integrative mechanisms of healthy ageing will not be best understood by using a patho-physiological approach that is systems based. This approach is slanted toward understanding and treating disease processes. Because exercise affects most physiological functions and processes (Morris and Heady, 1953; Nixon et al., 1976; Wilson and Tanaka, 2000; Heinonen et al., 2014; Sjøgaard et al., 2016; Duggal et al., 2018; Lazarus et al., 2018; Mailing et al., 2019; Joanisse et al., 2020; Kern et al., 2020), it is unlikely for healthy ageing to occur against the backdrop of an absence of physical activity. The effects of exercise on these processes are not monolithic (Sparks, 2017). Recently, four categories of regulation encompassing physiology, age and exercise have been proposed (Lazarus and Harridge, 2018; Lazarus et al., 2019). Category A consists of those processes that decreases by age alone, category B are those processes that are affected by age but can be modified by exercise, category C has processes that are unaffected by ageing but they too can be modified by exercise, and category D reside processes that are independent of both age and exercise. As people age the interactions of categories A, B, C, and D will be in constant flux (Lazarus et al., 2018). Thus, flow diagrams that depict any age-related changes in signaling or metabolic interactions are reflective of the state of physiology at a given age/moment in time.

The removal of exercise is an intervention that will impact negatively on all systems. As two categories of regulation (B and C), will be negatively affected directly by the absence of exercise, the overarching affect is that interactions between all four categories will be different between exercisers and non-exercisers. The same ageing process now interacting with a less optimal physiology produces the lifestyle diseases, whose incidence increases with age. These diseases can thus probably best be categorized as exercise deficiency diseases (Booth et al., 2012; Lazarus et al., 2018). While the development of disease may take time to manifest, it is possible to detect the markers of incipient disease relatively early in the sedentary.^1^ Non-exercisers may not be ill, but they are not healthy. It has been suggested that physical inactivity is the biggest health problem of the 21st century (Blair, 2009).

Heterogeneity is a ubiquitous characteristic of the presentation of both pathological and healthy human physiology (Chareonthaitawee et al., 2001; Aird, 2011; Lynch and Watt, 2018). Indeed, in health most systems will operate within reasonably well-defined constraints and have normative values (e.g., blood glucose, insulin, markers of liver function, etc.). While physiological functional and integrative indices can very markedly between individuals even when they show the similar physiognomy (e.g., in indices such as VO_2max_ and muscle function). The heterogeneity of phenotype is partly genetic but more generally reflects the underlying differences in the response and adaptation of physiological systems to external factors and in particular exercise (Walter et al., 2012; Lazarus and Harridge, 2018; Said et al., 2019). In non-exercisers, a lack of exercise imposes a different yet related heterogeneity; in that, all the same processes are now operating in an exercise deficient environment (Sparks, 2017). Although there is a very large literature of the role of heterogeneity in human disease and particularly in the generation of cancers (Klocke and Wittenberg, 1969; McClellan and King, 2010; Sominsky et al., 2020), there is sparse literature on the specific impact of heterogeneity on the presentation of human ageing. In a physiological spectrum ranging from healthy ageing, through sedentariness with its associated non-optimal physiology and continued into disease, indices may mirror the effects of this spectrum. For example, the range of VO_2max_ values produced by varying (e.g., through type, intensity, and duration) the exercise stimulus and the correlation of these values with individual health and disease markers is well documented (Blair et al., 1989; American College of Sports Medicine, 2014; Gries et al., 2018). The ageing process interacting with lifestyle factors and physiological heterogeneity, continuously and continually moderates and influences all physiological processes and systems. It is therefore legitimate to regard physiology as the science concerned with investigating the inherent ageing process. These points form the basis of an overarching hypothesis, which can be summarized as being that: when sufficient exercise is present, the multiple interactions of categories A, B, C, and D produce an ageing profile that remains essentially synchronous, coherent, and integrated throughout most of life. But, in the absence of sufficient exercise, physiology over time will deteriorate toward the production of a lifestyle disease. Thus, the ageing process interacts with the physiological heterogeneity present in categories A, B, C, and D, which will determine the age of presentation of a disease as well as the order of presentation of subsequent diseases. Below, we take three different groups of older individuals who differ in their exercise levels to expand on these points.

## Hypothesis: Integrating the Interplay of Ageing, Exercise, and Heterogeneity in Master Athletes

Master athletes represent a group of people who exercise regularly and intensely in later life (Ransdell et al., 2009; Rittweger et al., 2009) and at a point well over that required for healthy ageing (above their individual Set Point, Lazarus and Harridge, 2017). When performance times of running, cycling (Baker and Tang, 2010), and swimming (Donato et al., 2003) are followed over five decades, a clear pattern emerges. This pattern (essentially a curve with acceleration in the rate of decline during the eighth decade) is independent of sporting discipline and phenotype and applies to both men and women. The curves of decreasing performance time with age show no evidence of disruption by disease and were sufficiently similar for Baker and Tang (2010) to derive an equation that had a good fit for master running performance times across the complete spectrum of official distances available in flat races. The self-selection criteria for each discipline are rigorous. Athletes must have the body phenotype that is best suited to a given discipline are undertaking similar types of exercise training and at maximum training loads (commensurate with their age) as they seek to maximize their performance. In addition, their nutritional intake must be able to sustain their requirements both for energy expenditure and for the maintenance of healthy physiological function across all organs. While they also most probably have the necessary social and economic systems in place to allow them to keep them mentally focused on their tasks. When all these criteria are fulfilled, it has been hypothesized that these decreasing performance times are driven by the inherent human ageing process (Rittweger et al., 2009; Lazarus and Harridge, 2017). From the above, four important conclusions emerge. Firstly, these criteria remove the heterogeneity of decreases in performance times that might have been expected in a cross-sectional study (Donato et al., 2003). Secondly, the diversity of discipline and distance make no difference to the projection of the curves. Thirdly, the curve is independent of body shape and gender. Fourthly, this suggests that the human ageing process is probably the same in all humans (Lazarus and Harridge, 2017). This hypothesis is re-enforced by the fact that most people arrive at the same end point, i.e., death at around the end of the ninth decade (Office for National Statistics, UK, 2017).

If the ageing process is generating the shape of the performance curve, then any person who decides to participate in a discipline, in which training loads are at maximal for their physiological make up, should generate the same drop off in their performance curve if placed in competition (Lazarus and Harridge, 2018). Granted their times would be poor because they could be unsuited to the discipline chosen, but it is the ageing process that is driving the profile of the curves and not physical prowess.

It is important to realize that similar declining performance time curves need not necessarily be the resultant of the homogenization of the physiology of those taking part as many factors determine performance. It is the combination of these constituents, albeit at differing magnitudes, that determine final competitive outcomes. However, the ranges of magnitudes are confined within limits that are species dependent. Thus, the strict self-selection criteria result in a cohort of competitors who are not homogeneous physiologically, but who have a range of heterogeneities that are circumscribed to give similar performance times when subjected to exercise at maximum intensity.

## Hypothesis: Integrating the Interplay of Ageing, Exercise, and Heterogeneity in Habitual Exercisers

Having considered competitive athletes, the next step is to consider individuals who are very physically active and could be termed as “dedicated exercisers” (Bhella et al., 2014), but who are not competitive athletes. We recently undertook a well-controlled cross-sectional study on older cyclists (Pollock et al., 2015). Cyclists from 55 to 79 years were studied. In this study, volume of exercise, but not intensity, was measured. However, it was highly likely they were exercising at an exercise load that was sufficient, as indicated by their measured VO_2max_ values (American College of Sports Medicine, 2014), to offset the diseases associated with inactivity and above their set point for healthy ageing, but likely below that of a competitive athlete (Lazarus and Harridge, 2017). Under these conditions, an analysis of VO_2max_ data has shown that people of the same age could have markedly different VO_2max_ levels (Lazarus and Harridge, 2010; Pollock et al., 2015). The different VO_2max_ levels can be indicative of different exercise loads (Wilson and Tanaka, 2000), but other factors including genetics and specificity of training, are also at work causing differences between the same aged individuals and final VO_2max_ values (Magel et al., 1975; Bouchard et al., 1986; Noakes, 2003; Lundby et al., 2016). Thus, even at the same exercise training loads, heterogeneity in values can be demonstrated. Because of all these factors, it is not surprising that it was also shown that exercising people of different ages can exhibit the same VO_2max_ values (Pollock et al., 2015). These are important findings because they emphasize that health, as defined by being free of diseases due to lifestyle, can be maintained by a range of exercise loads that start at individual thresholds sufficient to counter sedentary diseases, their set points (Lazarus and Harridge, 2017) continue all the way up to maximum exercise loads. The physiological indices of all cyclists were exhibiting the sum of the spectrum of values produced by the dose dependent effect of exercise (Wilson and Tanaka, 2000; Bhella et al., 2014). All cross-sectional studies on relevant physiological indices will display this dose effect of exercise, and this will contribute to any inherent heterogeneity between individuals. Longitudinal studies on any individual, who is exercising above their set point, will over the period of study, show the heterogeneity of functions even if the absolute exercise load changes relative to age (Lambert et al., 2002). Thus, even when training at the same exercise loads relative to age, the ageing process will ensure that values of indices in the individual will fall.

Viewed from the perspectives stated above, the relation between biological age and chronological age carries a different connotation. A 70-year-old exercising at his or her set point (Lazarus and Harridge, 2017) is likely to be healthy, as defined by being free of the diseases of lifestyle and likely have a VO_2max_ value that will confirm that prediction (American College of Sports Medicine, 2014). The chronological age and biological ages of the 70-year-old are exactly where they should be for health. Now take another 70-year-old exercising at higher intensity. All other factors being equal, VO_2max_ will be higher. This 70-year-old is not more healthy or ageing more optimally but has a training-related enhancement of his/her cardio-respiratory fitness. In terms of biological health both individuals are matched because the dose threshold of exercise, necessary to ward off the diseases of inactivity, has been passed and the ageing trajectories (all other things being similar) no different.

A further point to consider on the subject of heterogeneity is that of differences in the tissue response to exercise within any given individual. While some organs will benefit directly from exercise (e.g., increased use of contracting muscle) others, such as the brain, also benefit, probably from the consequent increases in blood flow. One of the growing areas of interest is how exercise is beneficial to cognitive function during ageing (Northey et al., 2018). However, the rates at which adaptations to exercise occur or the rate at which different organs “age” under optimal circumstances remains to be determined.

## Hypothesis: Integrating the Interplay of Ageing and Heterogeneity in Non-Exercisers

As discussed, there are two regulatory mechanisms that are directly affected by exercise (Lazarus and Harridge, 2017; Lazarus et al., 2018). These are, firstly, category B, in which processes are age-affected but in which exercise can ameliorate the trajectory of change. Secondly, category C in which those mechanisms not affected by age but can be positively modified by exercise would also be adversely affected.

In non-exercisers interactions of categories A, B, C, and D will now be radically different from exercisers. For example, VO_2max_ levels will be lower across all ages when compared to exercising counterparts (Gries et al., 2018). If the development of disease is caused by falling outside normative threshold values, then the initial threshold to be broken will depend on the physiological make-up of the individual. Because of individual heterogeneity, it is not possible to predict which of the lifestyle-associated diseases will be the first to present. In addition, the presentation of a particular disease will not strictly follow ageing. It is not the ageing process that is the prime etiological factor underlying disease presentation, but the interaction of a universal ageing process on an exercise deficient physiology (Fried et al., 2001; Chiu and Wray, 2010; Lazarus et al., 2018).

## Hypothesis: The Interaction of Regulatory Categories A, B, C, and D with Exercise, Age, and Intrinsic Heterogeneity

**Figure 1:**
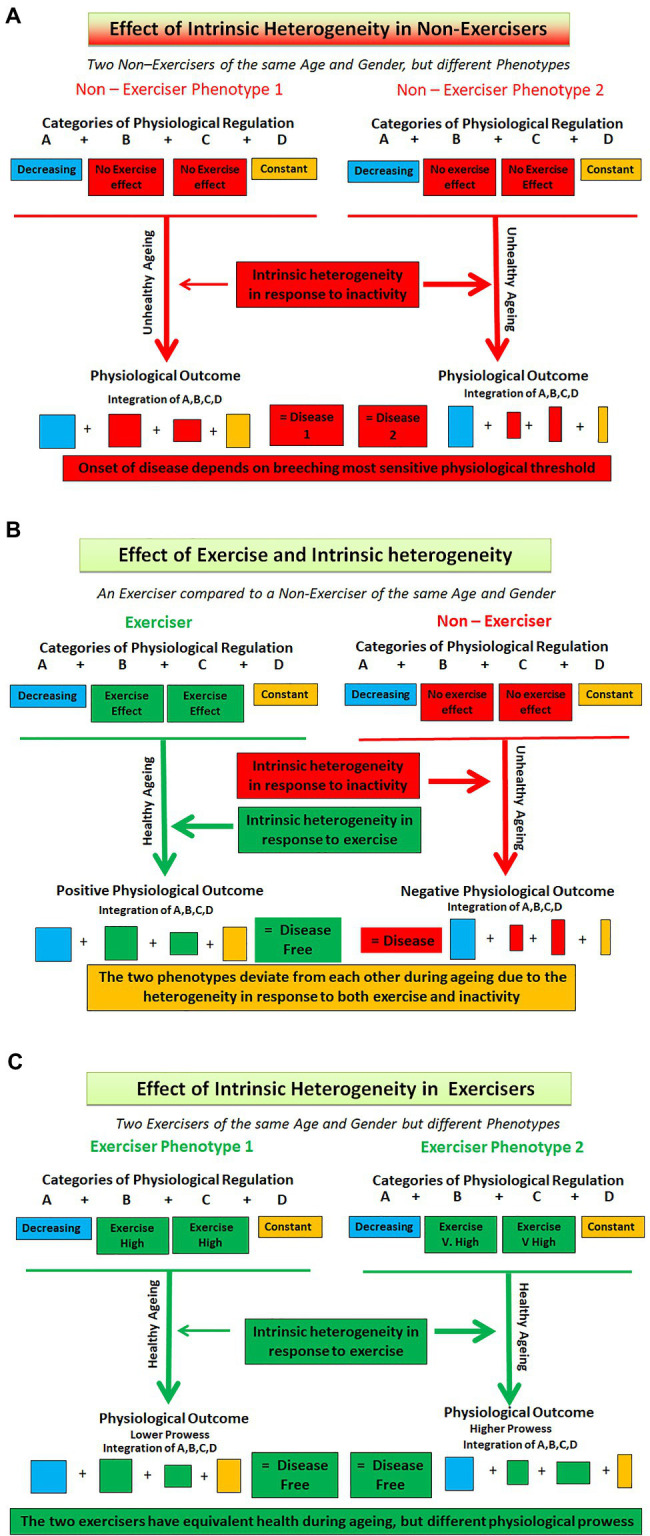
Depicting interaction of exercise and heterogeneity in exercisers and non – exercisers of the same age and gender. **(A)** Removal of exercise. Here a disease model is shown. Both individuals are non-exercisers, but different phenotypes are produced because of the effects of intrinsic heterogeneity in all categories and because Categories B and C are negatively affected by a lack of exercise. **(B)** Comparing the presence of exercise (Left hand side) with no exercise. Here the differences between the two individuals are not only due to exercise (Categories B and C) but also because of the intrinsic heterogeneity in the interactions of all four categories. **(C)** Here two exercisers undertaking the same levels of exercise are shown. Different phenotypes are produced because of the differing effects of exercise on categories B and C and because of the intrinsic heterogeneity and subsequent differences in the interactions of all four categories of regulation. The coloured boxes represent the four categories of regulation (A, B, C, and D), with the size of each box representing the heterogeneity within each category.

Healthy ageing is the product of the interaction of many factors. Firstly, there are the interactions of categories A, B, C, and D. These in turn can be modified. Categories A and B are age dependent and will therefore change over time. However, because category B is positively influenced by exercise the relationship between A and B will undergo further change. Category C that is independent of age is also modified by exercise. Category D is independent of age and exercise. However, overlying all these different properties of the categories is inherent human physiological heterogeneity. We have depicted this schematically in Figure 1. Thus, the interaction of age, exercise, and human heterogeneity will ensure that the relationships between processes residing in any of the four categories will be undergoing continuous change both in them and in relation to their interactions with other categories. All these processes are operating on a global scale and affect all physiological systems. To seek a single index, which will define the mechanism of these effects across the whole of physiology, borders on the futile. There is no magic index, as has been postulated for some diseases (Jackson and Schoenwaelder, 2003; Benfeito et al., 2013; Venkata and Ram, 2019), that will suffice as the definitive marker of healthy ageing.

## Healthy and Non-Healthy Phenotypes

The removal of exercise will affect two of the four regulatory categories. The outcomes of the regulatory interaction will begin to diverge between exercisers and non-exercisers from the time that exercise is removed. In exercisers the outcome, over time, is a physiology that is coherent and integrated while shrinking under the influence of age. In non-exercisers the outcome is eventually disease (Figure 1). Physiological regulation is now geared to preserving function in the face of a disease process. The regulatory mechanisms underpinning the two phenotypes will be following different trajectories. The physiology of non-exercisers should not be used as exemplars of health. All physiological parameters measured in this group of people will probably represent either incipient ill health at the best or disease at the worst. By the same token, the addition of exercise to a previously inactive scenario will have positive effects on the two categories and thus the regulation and interaction of all four. The known improvements in physiological and clinical indices as a result of exercise training interventions are well-known. However, what remains to be determined is at what age category A indices have diminished such that the interventions have come too late to be able to put an individual on a track that would match their optimal ageing trajectory.

## Summary

This hypothesis article has attempted to provide a logical foundation for unraveling the multifaceted and evolving conundrum that is the human ageing process. We have proposed that the decline in physiological function is determined by four interacting categories of regulation which overtime manifests itself as a continually changing physiological landscape. These categories have the role of exercise and an acknowledgement of the heterogeneity as core components. Healthy ageing, defined here as being free from diseases of lifestyle, only occurs when exercising at set point and above. The investigation of master athletes who are at the maximum end of that exercising spectrum will give no greater insight into the mechanisms underlying the healthy human ageing process than investigating those exercising at set point. It is not possible to be healthier than healthy.

There is no evidence that an increased proficiency of exercise discipline alters the health trajectory of any individual. However, master athletes will give insight into the effects of age on performance and by default on integrative physiological function. The removal of exercise causes a disintegration of ageing physiology such that disease is an almost inevitable outcome. In all cross-sectional studies only a snapshot of the effect of age, exercise, and heterogeneity on physiological indices and systems is obtained. Over time, exercise and heterogeneity interact, causing constant flux in complex biochemical, molecular, and physiological processes. A healthy, exercising 70-year-old not only differs from a healthy exercising 50-year-old in chronological age but also in having a physiology that has had to adapt to ageing and lifestyle interactions, so that integrity, co-ordination, and synchronization are maintained by a shrinking physiology. How this integrity is maintained awaits discovery. The human ageing process, interacting with lifestyle factors and heterogeneous physiologies, governs and modifies all processes in all humans all the time, yet its presence does not seem to merit a place in most physiology or medical texts.

## Author Contributions

Both authors listed have made a substantial, direct and intellectual contribution to the work, and approved it for publication.

## Funding

Funding from Nadace The JetBrains Foundation is acknowledged.

## Conflict of Interest

The authors declare that the research was conducted in the absence of any commercial or financial relationships that could be construed as a potential conflict of interest.

## Publisher’s Note

All claims expressed in this article are solely those of the authors and do not necessarily represent those of their affiliated organizations, or those of the publisher, the editors and the reviewers. Any product that may be evaluated in this article, or claim that may be made by its manufacturer, is not guaranteed or endorsed by the publisher.

## Footnotes

^1^ www.nhlbi.nih.gov
